# Dai-Kou type conjugate gradient methods with a line search only using gradient

**DOI:** 10.1186/s13660-017-1341-z

**Published:** 2017-04-04

**Authors:** Yuanyuan Huang, Changhe Liu

**Affiliations:** School of Mathematics and Statistics, Henan University of Science and Technology, Luoyang, 471023 P.R. China

**Keywords:** conjugate gradient, optimality condition, line search, sufficient descent condition, global convergence

## Abstract

In this paper, the Dai-Kou type conjugate gradient methods are developed to solve the optimality condition of an unconstrained optimization, they only utilize gradient information and have broader application scope. Under suitable conditions, the developed methods are globally convergent. Numerical tests and comparisons with the PRP+ conjugate gradient method only using gradient show that the methods are efficient.

## Introduction

Consider the following problem of finding $x\in R^{n}$ such that

1 $$ g(x)=0, $$

where $g:R^{n}\rightarrow R^{n}$ is continuous. Throughout this paper, problem (1) corresponds to the first-order optimality condition of the unconstrained optimization

2 $$ \min f(x), $$

where $f:R^{n}\rightarrow R$ is the function whose gradient is *g*.

Conjugate gradient methods are very efficient in solving large scale problem (2), if *f* is known, due to their simple iteration and their low memory requirements. For any given starting point $x_{0}\in R^{n}$, an iterative sequence $\{ x_{k}\}$ is generated by the following form:

3 $$ x_{k+1}=x_{k}+\alpha_{k} d_{k}, $$

where $\alpha_{k}$ is a step-length obtained by some line search, and $d_{k}$ is a search direction generated by

4 $$ d_{k}= \textstyle\begin{cases} -g_{k}, &\text{if }k=0, \\ -g_{k}+\beta_{k} d_{k-1}, &\text{if }k\geq1, \end{cases} $$

where $g_{k}=g(x_{k})$. Different choices of the parameter $\beta_{k}$ in (4) lead to different nonlinear conjugate gradient methods. The Fletcher-Reeves [1], Hestenes-Stiefel [2], Polak-Ribiére-Polyak [3, 4], Dai-Yuan [5] and Liu-Storey [6] formulas, and so on, are well-known formulas for $\beta_{k}$. Particularly, conjugate gradient methods with the following (sufficient) descent condition

5 $$ g_{k}^{T} d_{k}\leq-c \|g_{k}\|^{2}, \quad \forall k\geq0, c>0, $$

are very important and are always more efficient.

Recently, Dai and Kou [7] designed a family of conjugate gradient methods for the unconstrained nonlinear problems, the corresponding search direction is close to the direction of the scaled memoryless BFGS method. More importantly, they satisfied the sufficient descent condition (5). Numerical experiments illustrated that the Dai-Kou type conjugate gradient methods are more efficient than the Hager-Zhang type methods [8] presented by Hager and Zhang [8, 9]. For other descent conjugate gradient methods proposed by researchers, please see [7, 9–11] and the references therein.

For conjugate gradient methods, line search plays an important role for the global convergence. In general, the weak Wolfe line search,

6 $$\begin{aligned}& f(x_{k}+\alpha_{k} d_{k}) \leq f(x_{k})+\delta\alpha_{k} g_{k}^{T} d_{k}, \end{aligned}$$

7 $$\begin{aligned}& \sigma g_{k}^{T} d_{k} \leq g(x_{k}+ \alpha_{k} d_{k})^{T} d_{k}, \end{aligned}$$

where $0<\delta<\sigma<1$, was used to obtain the step-length $\alpha _{k}$. Hager and Zhang [9] showed that the first condition (6) may never be satisfied due to the existence of the numerical errors (see also [7]). Thus, in order to avoid the numerical drawback of the weak Wolfe line search, they proposed approximate Wolfe conditions [8, 9], which was a combination of the weak Wolfe line search and

8 $$ \sigma g_{k}^{T} d_{k}\leq g(x_{k}+\alpha_{k} d_{k})^{T} d_{k}\leq(2\delta-1) g_{k}^{T} d_{k}, $$

where $0<\delta<1/2$ and $\delta<\sigma<1$. Numerical tests showed that the combined line search performed well, but there is no theory to guarantee the global convergence. Then Dai and Kou proposed an improved Wolfe line search, that is, the step-length $\alpha_{k}$ satisfied (7) and

9 $$ f(x_{k}+\alpha_{k} d_{k})\leq f(x_{k})+\min\bigl\{ \epsilon \bigl\vert g_{k}^{T} d_{k} \bigr\vert , \delta \alpha_{k} \bigl\vert g_{k}^{T} d_{k} \bigr\vert +\eta_{k} \bigr\} , $$

where $0<\delta<\sigma<1$, $\epsilon>0$ is a constant parameter and $\{\eta_{k}\}$ is a positive sequence satisfying $\sum_{k\geq0}\eta_{k}<+\infty$. With the improved Wolfe line search, the global convergence of Dai-Kou type conjugate gradient methods was guaranteed.

Although the Hager-Zhang type and Dai-Kou type conjugate gradient methods are efficient in solving problem (2), during the implementation of the methods, function evaluations are required. The goal of this paper is to solve problem (1) which is more general and includes some nonlinear equations, such as boundary value problems [12]. So, we hope to improve the Dai-Kou type conjugate gradient methods to directly solve problem (1) and retain their high numerical efficiency. More recently, Dong [13] embedded an Armijo-type line search only using gradient into the PRP+ conjugate gradient method [14] to solve problem (1), the step-length $\alpha_{k}$ satisfied

10 $$ g(x_{k}+\alpha_{k} d_{k})^{T} d_{k}+\frac{1}{2}\max\{-\mu_{k},0\} \alpha_{k}\| d_{k}\|^{2}\leq\sigma g_{k}^{T} d_{k}, $$

where $\mu_{k}$ is a determined real number and $0<\sigma<1$. The line search allowed small choices of $\alpha_{k}$. In order to avoid this drawback, Dong [15] considered the following line search:

11 $$ \sigma g_{k}^{T} d_{k} \leq g(x_{k}+\alpha_{k} d_{k})^{T} d_{k} \leq\delta g_{k}^{T} d_{k}, $$

where $0<\delta<\sigma<1$. Motivated by the work of [15], we embed the line search (11) into the Dai-Kou type conjugate gradient methods, then the improved methods of this paper have several advantages. They have the positive features of the Dai-Kou type methods for problem (2), they can be used to solve the nonlinear optimization (2) only requiring gradient information, and they can be used to solve some systems of nonlinear equations, such as those arising in boundary value problems and others.

The rest of this paper is organized as follows. In the next section, we simply review the Dai-Kou type conjugate gradient methods for unconstrained minimization and develop them to solve problem (1). In Section 3, we prove the global convergence of the improved methods under some suitable conditions. In Section 4, we select two classes of test problems to test the improved methods. One class is composed of test problems from the CUTEst test environment, and the other class is composed of some boundary value problems. The numerical performance is used to confirm their broader application and to compare with that of the PRP+ conjugate gradient method in [13]. Finally, some conclusions are given in Section 5.

## Algorithm

In this section, we describe the details of the proposed methods. First, we briefly review the Dai-Kou type conjugate gradient methods in the setting of unconstrained minimization (2). We have mentioned above that nonlinear conjugate gradient methods are identified by the definitions of the parameter $\beta_{k}$ in (4). For the family of Dai-Kou type conjugate gradient methods, the parameter $\beta_{k}$ is defined as

12 $$ \beta_{k}^{N}(\tau_{k-1})=\max \biggl\{ \beta_{k}(\tau_{k-1}),\eta \frac{g_{k}^{T} d_{k-1}}{\|d_{k-1}\|^{2}} \biggr\} . $$

Here,

13 $$ \beta_{k}(\tau_{k-1})=\frac{g_{k}^{T} y_{k-1}}{d_{k-1}^{T} y_{k-1}}- \biggl(\tau_{k-1}+\frac{\|y_{k-1}\|^{2}}{s_{k-1}^{T} y_{k-1}}-\frac{s_{k-1}^{T} y_{k-1}}{\|s_{k-1}\|^{2}} \biggr) \frac{g_{k}^{T} s_{k-1}}{d_{k-1}^{T} y_{k-1}}, $$

where $y_{k-1}=g_{k}-g_{k-1}$, $s_{k-1}=\alpha _{k-1}d_{k-1}=x_{k}-x_{k-1}$, $\tau_{k-1}$ is a parameter corresponding to the scaling parameter in the scaled memoryless BFGS method, and $\eta\in[0,1)$. The parameters $\beta_{k}$ in the Dai-Liao type methods [16] and the Hager-Zhang type methods [9] are special cases of formula (13). If $\tau _{k-1}$ is specially defined as

14 $$ \tau_{k-1}=\lambda\tau_{k-1}^{A}+(1- \lambda)\tau_{k-1}^{B} $$

with $\lambda\in[0,1]$ and

15 $$\begin{aligned}& \tau_{k-1}^{A} = \frac{\|y_{k-1}\|^{2}}{s_{k-1}^{T} y_{k-1}}, \end{aligned}$$

16 $$\begin{aligned}& \tau_{k-1}^{B} = \frac{s_{k-1}^{T} y_{k-1}}{\|s_{k-1}\|^{2}}, \end{aligned}$$

then the Dai-Kou type conjugate gradient methods satisfy the sufficient descent condition (5).

The Dai-Kou type methods are very efficient in solving the unconstrained minimization, so we hope they can be used to solve problem (1) only requiring gradient information. Now we describe the improved methods in detail.

### Algorithm 2.1

Step 0.: Choose $x_{0}\in R^{n}$, constants $\sigma\in(0,1)$, $\delta\in (0,\sigma)$, $\lambda\in[0,1]$, $\eta\in[0,1)$, $\varepsilon>0$. Set $g_{0}:=g(x_{0})$ and $k:=0$.
Step 1.: If $\|g_{k}\|_{\infty}\leq\varepsilon$, then stop.
Step 2.: Generate the search direction $d_{k}$ by (4) with $\beta_{k}$ from (12), where $\tau_{k-1}$ is defined by (14).
Step 3.: Find $\alpha_{k}$ such that condition (11) holds, then compute the new iterate $x_{k+1}=x_{k}+\alpha_{k} d_{k}$. Set $k:=k+1$ and go to Step 1.

In Step 3, the step-length $\alpha_{k}$ is determined following the inexact line search strategies of Algorithm 2.6 in [17]. Detailed steps are described in the following line search algorithm.

### Algorithm 2.2

Step 0.: Set $u=0$ and $v=+\infty$. Choose $\alpha>0$. Set $j:=0$.
Step 1.: If *α* does not satisfy
  $$g(x_{k}+\alpha d_{k})^{T} d_{k}\leq \delta g_{k}^{T} d_{k}, $$
  then set j:=j+1, and go to Step 2. If *α* does not satisfy
  $$\sigma g_{k}^{T} d_{k}\leq g(x_{k}+ \alpha d_{k})^{T} d_{k}, $$
  then set $j:=j+1$, and go to Step 3. Otherwise, set $\alpha_{k}:=\alpha$, and return.
Step 2.: Set $v=\alpha$, $\alpha=(u+v)/2$. Then go to Step 1.
Step 3.: Set $u=\alpha$, $\alpha=2u$. Then go to Step 1.

The choice of the initial step-length is important for a line search. For conjugate gradient methods, it is important to make an initial guess of the step-length by utilizing the current iterative information about the problem. So, in Algorithm 2.2, we choose the initial step-length $\alpha=1/\|g_{0}\|$ if $k=0$, and $\alpha=\alpha _{k-1}g_{k-1}^{T} d_{k-1}/y_{k-1}^{T} d_{k-1}$ if $k\geq1$.

## Convergence analysis

### Assumption 1

Assume that $f: R^{n}\rightarrow R$ is bounded below, that is, $f(x)>-\infty$ for all $x\in R^{n}$, and *f* is continuously differentiable. Its gradient $g: R^{n}\rightarrow R^{n}$ is *L*-Lipschitz continuous, that is, there exists a constant $L>0$ such that

17 $$ \bigl\Vert g(x)-g(y) \bigr\Vert \leq L \Vert x-y \Vert ,\quad \forall x, y\in R^{n}. $$

Assumption 1 implies that there exists a positive constant *γ̂* such that

18 $$ \bigl\Vert g(x) \bigr\Vert \leq\hat{\gamma}, \quad \forall x\in R^{n}. $$

### Lemma 3.1

*Assume that* $g: R^{n}\rightarrow R^{n}$ *satisfies Assumption* 1. *If* $d_{0}=-g_{0}$ *and* $d_{k-1}^{T} y_{k-1} \neq0$ *for all* $k \geq1$, *then*

19 $$ g_{k}^{T} d_{k}\leq-\min \biggl\{ \frac{3}{4},1-\eta \biggr\} \|g_{k}\|^{2}. $$

### Proof

Since $d_{0}=-g_{0}$, we have $g_{0}^{T} d_{0}=-\|g_{0}\|^{2}$, which satisfies (19). If

$$ \beta_{k}^{N}(\tau_{k-1})=\frac{g_{k}^{T} y_{k-1}}{d_{k-1}^{T} y_{k-1}}- \biggl(\tau_{k-1}+\frac{\|y_{k-1}\|^{2}}{s_{k-1}^{T} y_{k-1}}-\frac {s_{k-1}^{T} y_{k-1}}{\|s_{k-1}\|^{2}}\biggr) \frac{g_{k}^{T} s_{k-1}}{d_{k-1}^{T} y_{k-1}}, $$

from Lemma 2.3 in [5], we have the result that

$$ g_{k}^{T} d_{k}\leq-\frac{3}{4} \|g_{k}\|^{2}. $$

And if

$$ \beta_{k}^{N}(\tau_{k-1})=\eta\frac{g_{k}^{T} d_{k-1}}{\|d_{k-1}\|^{2}}, $$

it is easy to know that

$$ g_{k}^{T} d_{k}\leq-(1-\eta)\|g_{k} \|^{2}. $$

The proof is complete. □

### Lemma 3.2

*Suppose that* $f:R^{n}\rightarrow R$ *is bounded below along the ray* $\{ x_{k}+\alpha d_{k} | \alpha>0\}$, *its gradient* *g* *is continuous*, $d_{k}$ *is a search direction at* $x_{k}$, *and* $g_{k}^{T} d_{k}<0$. *Then if* $0<\delta <\sigma<1$, *there exists* $\alpha_{k}>0$ *satisfying the line search* (11).

### Proof

Define $\phi(\alpha)=f(x_{k}+\alpha d_{k})$ and $\psi(\alpha )=f(x_{k})+\alpha\delta g_{k}^{T} d_{k}$. Since $\phi(\alpha)$ is bounded below for all $\alpha>0$, $0<\delta <1$ and $g_{k}^{T} d_{k}<0$, the functions $\phi(\alpha)$ and $\psi(\alpha )$ must intersect at at least one point. Let $\alpha_{k}^{*}>0$ be the smallest intersecting value of *α*, i.e.,

20 $$ f\bigl(x_{k}+\alpha_{k}^{*} d_{k} \bigr)=f(x_{k})+\alpha_{k}^{*} \delta g_{k}^{T} d_{k}. $$

Since *f* is continuously differentiable, by the mean value theorem, there exists $\alpha_{k}\in(0,\alpha_{k}^{*})$ such that

21 $$ f\bigl(x_{k}+\alpha_{k}^{*} d_{k} \bigr)-f(x_{k})=\alpha_{k}^{*} g(x_{k}+ \alpha_{k} d_{k})^{T} d_{k}. $$

By combining (20) and (21), we obtain

22 $$ \delta g_{k}^{T} d_{k}=g(x_{k}+ \alpha_{k} d_{k})^{T} d_{k}. $$

Furthermore,

23 $$ \sigma g_{k}^{T} d_{k}\leq g(x_{k}+\alpha_{k} d_{k})^{T} d_{k}= \delta g_{k}^{T} d_{k}, $$

since $0<\delta<\sigma<1$ and $g_{k}^{T} d_{k}<0$. □

### Lemma 3.3

*Assume that* $g:R^{n}\rightarrow R^{n}$ *is monotone on the interval* $\{ x_{k}+\alpha d_{k} : 0 \leq\alpha\leq\alpha_{k}\}$, *where* $\alpha_{k}$ *satisfies the line search* (11), *then the following inequality holds*:

24 $$ f(x_{k}+\alpha_{k} d_{k})\leq f(x_{k})+\delta\alpha_{k} g_{k}^{T} d_{k}, $$

*where* $f:R^{n}\rightarrow R$ *is the function whose gradient is* *g*.

### Proof

Since *g* is monotone on the interval $\{x_{k}+\alpha d_{k} : 0 \leq\alpha \leq\alpha_{k}\}$, then

$$ \bigl(g(x_{k}+\alpha_{k} d_{k})-g(x_{k}+ \alpha d_{k})\bigr)^{T} \bigl((x_{k}+ \alpha_{k} d_{k})-(x_{k}+\alpha d_{k}) \bigr)\geq0. $$

Since $\alpha\leq\alpha_{k}$, it is not difficult to get that

$$g(x_{k}+\alpha d_{k})^{T} d_{k} \leq g(x_{k}+\alpha_{k} d_{k})^{T} d_{k}\leq\delta g_{k}^{T} d_{k}. $$

Applying this inequality to the following relation

$$f(x_{k}+\alpha_{k} d_{k})=f(x_{k})+ \int_{0}^{\alpha_{k}} g(x_{k}+\alpha d_{k})^{T} d_{k} \, d\alpha $$

yields inequality (24). □

Now, we state the Zoutendijk condition [18] for the line search (11).

### Lemma 3.4

*Assume that* $g:R^{n}\rightarrow R^{n}$ *satisfies Assumption* 1. *Consider any iterative method in the form* (3), *where* $d_{k}$ *is a descent direction and* $\alpha_{k}$ *satisfies the line search* (11), *then*

25 $$ \sum_{k\geq0}\frac{(g_{k}^{T} d_{k})^{2}}{\|d_{k}\|^{2}}< +\infty. $$

### Proof

It follows from the Cauchy-Schwarz inequality, the Lipschitz condition (17) and the line search (11) that

26 $$ (\sigma-1)g_{k}^{T} d_{k} \leq(g_{k+1}-g_{k})^{T} d_{k}\leq \alpha_{k} L \|d_{k}\|^{2}. $$

Then we have

27 $$ \alpha_{k}\geq\frac{1-\sigma}{L} \frac{-g_{k}^{T} d_{k}}{\|d_{k}\|^{2}}. $$

The formula with (24) implies that

28 $$ \frac{(g_{k}^{T} d_{k})^{2}}{\|d_{k}\|^{2}}\leq\frac{L}{(1-\sigma)\delta }\bigl(f(x_{k})-f(x_{k+1}) \bigr). $$

Summing (28) over *k* and noting that *f* is bounded below, we have that the desired result holds. □

Now we discus the convergence properties of Algorithm 2.1. In the following, we will prove that if the gradient $g:R^{n}\rightarrow R^{n}$ is *μ*-strongly monotone, that is, there exists a constant $\mu >0$ such that

29 $$ \bigl(g(x)-g(y)\bigr)^{T} ( x-y ) \geq\mu\|x-y\|^{2},\quad \forall x, y\in R^{n}, $$

Algorithm 2.1 is globally convergent with $\lim_{k\rightarrow \infty}\|g_{k}\|=0$, and for more general gradient $g:R^{n}\rightarrow R^{n}$, Algorithm 2.1 is convergent in the sense that $\liminf_{k\rightarrow\infty}\|g_{k}\|=0$.

### Theorem 3.1

*Assume that* $g:R^{n}\rightarrow R^{n}$ *satisfies Assumption* 1 *and is* *μ*-*strongly monotone*. *The sequence* $\{x_{k}\}$ *is generated by Algorithm* 2.1, *then*

30 $$ \lim_{k\rightarrow\infty}\|g_{k}\|=0. $$

### Proof

It follows from (17) and (29) that

31 $$\begin{aligned}& s_{k-1}^{T} y_{k-1}\leq\|s_{k-1}\| \|y_{k-1}\| \leq L\|s_{k-1}\| ^{2}, \end{aligned}$$

32 $$\begin{aligned}& \mu\|s_{k-1}\|^{2} \leq s_{k-1}^{T} y_{k-1}. \end{aligned}$$

By (31) and (32), it is easy to see that

33 $$\begin{aligned}& \frac{s_{k-1}^{T} y_{k-1}}{\|s_{k-1}\|^{2}} \leq L, \end{aligned}$$

34 $$\begin{aligned}& \frac{\|y_{k-1}\|^{2}}{s_{k-1}^{T} y_{k-1}} \leq \frac{L^{2}}{\mu }. \end{aligned}$$

Then we have that

$$|\tau_{k-1}|\leq(1-\lambda)\frac{L^{2}}{\mu}+\lambda L. $$

Consequently, we have that

$$\begin{aligned} \bigl\vert \beta_{k}(\tau_{k-1}) \bigr\vert =& \biggl\vert \frac{g_{k}^{T} y_{k-1}}{d_{k-1}^{T} y_{k-1}}-\biggl(\tau_{k-1}+\frac{\|y_{k-1}\|^{2}}{s_{k-1}^{T} y_{k-1}}- \frac {s_{k-1}^{T} y_{k-1}}{\|s_{k-1}\|^{2}}\biggr)\frac{g_{k}^{T} s_{k-1}}{d_{k-1}^{T} y_{k-1}} \biggr\vert \\ \leq& \biggl[\frac{(2-\lambda)L^{2}}{\mu^{2}}+\frac{(2+\lambda )L}{\mu} \biggr]\frac{\|g_{k}\|}{\|d_{k-1}\|}. \end{aligned}$$

Furthermore,

$$ \bigl\vert \beta^{N}_{k}(\tau_{k-1}) \bigr\vert \leq\max \biggl\{ \frac{(2-\lambda )L^{2}}{\mu^{2}}+\frac{(2+\lambda)L}{\mu},\eta \biggr\} \frac{\|g_{k}\| }{\|d_{k-1}\|}. $$

Then

35 $$\begin{aligned} \Vert d_{k} \Vert =& \bigl\Vert -g_{k}+ \beta^{N}_{k}(\tau_{k-1})d_{k-1} \bigr\Vert \\ \leq& \Vert g_{k} \Vert + \bigl\vert \beta^{N}_{k}( \tau_{k-1}) \bigr\vert \Vert d_{k-1} \Vert \\ \leq&\zeta \Vert g_{k} \Vert , \end{aligned}$$

where $\zeta=1+\max\{\frac{(2-\lambda)L^{2}}{\mu^{2}}+\frac {(2+\lambda)L}{\mu},\eta\}$.

By Lemmas 3.1 and 3.4, we have that

$$\sum_{k\geq0}\frac{\|g_{k}\|^{4}}{\|d_{k}\|^{2}}< \infty. $$

It follows from this and (35) that

$$\sum_{k\geq0}\|g_{k}\|^{2}< \infty, $$

which implies the desired result. □

### Theorem 3.2

*Assume that* $g:R^{n}\rightarrow R^{n}$ *satisfies Assumption* 1. *Then Algorithm* 2.1 *is convergent in the sense that*

36 $$ \liminf_{k\rightarrow\infty} \|g_{k}\|=0. $$

### Proof

We prove the theorem by contradiction. Assume that both $g_{k}\neq0$ for all *k* and $\liminf_{k\rightarrow\infty} \|g_{k}\|>0$, then there must exist some $\gamma>0$ such that

37 $$ \|g_{k}\|\geq\gamma,\quad \forall k\geq0, $$

then $d_{k}\neq0$, otherwise Lemma 3.1 would imply $g_{k}=0$.

It follows from (37), Lemma 3.1 and Lemma 3.4 that

$$ \gamma^{2}\sum_{k\geq0} \frac{1}{\|d_{k}\|^{2}}\leq\sum_{k\geq0}\frac {\|g_{k}\|^{2}}{\|d_{k}\|^{2}} $$

and

38 $$ \sum_{k\geq0}\frac{\|g_{k}\|^{2}}{\|d_{k}\|^{2}}\leq\sum _{k\geq0}\frac {1}{\gamma^{2} }\frac{\|g_{k}\|^{4}}{\|d_{k}\|^{2}}\leq \frac{1}{\gamma^{2} \bar{c}}\sum_{k\geq0}\frac{(g_{k}^{T} d_{k})^{2}}{\|d_{k}\|^{2}}< \infty, $$

where $\bar{c}=\min\{\frac{3}{4},1-\eta\}$, then we have that

39 $$ \|d_{k}\|\rightarrow+\infty. $$

This means that there exists a positive integer *N*, for all $k\geq N$,

40 $$\begin{aligned} \beta_{k}^{N}(\tau_{k-1}) =& \beta_{k}(\tau_{k-1}) \\ =& \frac{g_{k}^{T} y_{k-1}}{d_{k-1}^{T} y_{k-1}}- \biggl(\tau_{k-1}+\frac {\|y_{k-1}\|^{2}}{s_{k-1}^{T} y_{k-1}}- \frac{s_{k-1}^{T} y_{k-1}}{\|s_{k-1}\| ^{2}} \biggr)\frac{g_{k}^{T} s_{k-1}}{d_{k-1}^{T} y_{k-1}} \\ =& \frac{g_{k}^{T} y_{k-1}}{d_{k-1}^{T} y_{k-1}}- \biggl((1+\lambda)\frac {\|y_{k-1}\|^{2}}{s_{k-1}^{T} y_{k-1}}-\lambda \frac{s_{k-1}^{T} y_{k-1}}{\| s_{k-1}\|^{2}} \biggr)\frac{g_{k}^{T} s_{k-1}}{d_{k-1}^{T} y_{k-1}}. \end{aligned}$$

It follows from Lemma 3.1, (11) and (37) that

41 $$ d_{k-1}^{T} y_{k-1}\geq-(1- \sigma)g_{k-1}^{T} d_{k-1}\geq\bar{c} (1-\sigma) \gamma^{2}. $$

It follows from (18), (33), (34), (40), (41) and the *L*-Lipschitz continuity of *g* that, for all $k\geq N$,

42 $$ \bigl\vert \beta_{k}^{N}( \tau_{k-1}) \bigr\vert \leq\frac{\hat{\gamma}(1+\lambda)}{\bar{c}(1-\sigma)\gamma^{2}}\biggl(L+ \frac{L^{2}}{\mu}\biggr)\|s_{k-1}\|. $$

Define $u_{k}=d_{k}/\|d_{k}\|$, then similarly to the proof of Lemma 4.3 in [7], we can get the result that

43 $$ \|u_{k}-u_{k-1}\|\leq2(1+\eta) \frac{\|g_{k}\|}{\|d_{k}\|}. $$

Then it follows from (38) and (43) that

44 $$ \sum_{k\geq1}\|u_{k}-u_{k-1} \|^{2}< \infty. $$

From Assumption 1 and Lemma 3.3, we know that the generated sequence $\{x_{k}\}$ is bounded, then there exists some positive constant *γ̄* such that

45 $$ \|x_{k}\|\leq\bar{\gamma},\quad \forall k \geq0. $$

By using inequalities (42), (44) and (45), we can get the desired result similarly to the proof of items II and III of Theorem 3.2 in [9]. □

## Numerical experiments

In this section, we did some numerical experiments to test the performance of the proposed method and compared it with the PRP+ conjugate gradient method in [13]. All codes were written in Matlab and run on a notebook computer with an Intel(R) Core(TM) i5-5200U 2.20 GHz CPU, 8.00 GB of RAM and Linux operation system Ubuntu 12.04. All test problems were drawn from the CUTEst test library [19, 20] and the literature [12]. For the test problems from the CUTEst test library, we particularly chose the unconstrained optimization problems whose dimensions were at least 50. Different from the work in the literature such as [5, 7], we solved them only using gradient information. In order to confirm the broader application scope of the proposed method, some boundary value problems were selected from [12]. See Chapter 1 in [21] for the background of the boundary value problems.

In practical implementations, the stopping criterion used was $\|g_{k}\|_{\infty}\leq10^{-3}$. For the proposed method in this paper, the values of *σ* and *δ* in the line search (11) were taken to be 0.9 and 0.0001, respectively, $\lambda=0.5$, and $\eta=0.5$. For the PRP+ conjugate gradient, all the initial values came from the reference [13].

**Figure 1:**
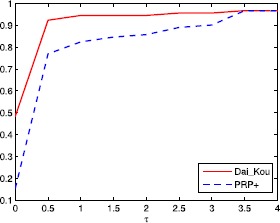
**Performance profile for the test problems from the CUTEst library based on the number of iterations.**

**Figure 2:**
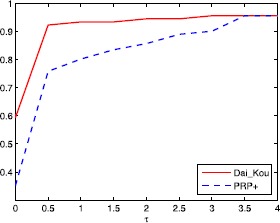
**Performance profile for the test problems from the CUTEst library based on the number of gradient evaluations.**

**Figure 3:**
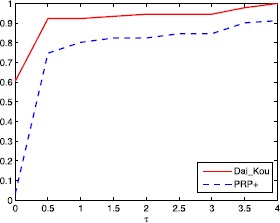
**Performance profile for the test problems from the CUTEst library based on the CPU time.**

**Figure 4:**
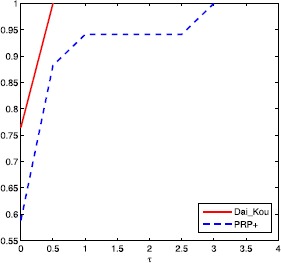
**Performance profile for some boundary value problems based on the number of iterations.**

**Figure 5:**
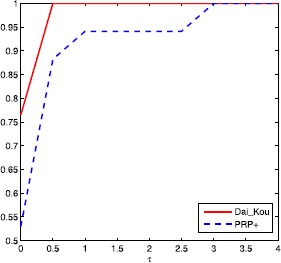
**Performance profile for some boundary value problems based on the number of gradient evaluations.**

**Figure 6:**
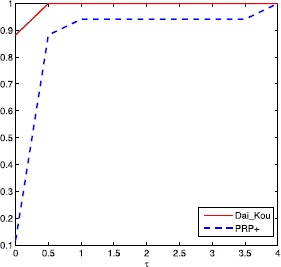
**Performance profile for some boundary value problems based on the CPU time.**

The numerical results are reported in Tables 1 and 2, where Name, Dim, Iter, Ng and CPU represent the name of the test problem, the dimension, the number of iterations, the number of gradient evaluations and the CPU time elapsed in seconds, respectively. ‘-’ means the method failed to achieve the prescribed accuracy when the number of iterations exceeded 50,000 or the gradient function generated ‘NaN’. The performances of the two methods were evaluated using the profiles of Dolan and Morè [22]. That is, we plotted the fraction P of the test problems for which each of the two methods was within a factor *τ*. In the performance profiles, the top curve represents the most robust one within the same factor *τ*, and the left curve represents the fastest one to solve the same percentage of test problems. Figures 1-3 show the performance profiles for test problems from the CUTEst library relating to the number of iterations, the number of gradient evaluations and the CPU time, respectively. Figures 4-6 show the performance profiles for some boundary value problems. These figures reveal that, for the test problems, the proposed method is more efficient and robust than the PRP+ conjugate gradient method. Consequently, the improved method not only can solve problems only referring to gradient information but also inherits the good numerical performance of the Dai-Kou type conjugate gradient methods.

**Table 1:** **Numerical results for test problems from the CUTEst library**

**Name (Dim)**	**Method**	**Iter/Ng/CPU**
ARGLINA (200)	Dai_Kou	14/28/1.673e − 02
	PRP+	13/25/2.309e − 02
ARGLINB (200)	Dai_Kou	22 /43/2.577e − 02
	PRP+	47/93/6.121e − 02
ARGLINC (200)	Dai_Kou	22/43/2.420e − 02
	PRP+	47/92/6.144e − 02
BDQRTIC (500)	Dai_Kou	118/264/3.731e − 02
	PRP+	181/317/6.208e − 02
BOX (10,000)	Dai_Kou	30/100/1.662e − 01
	PRP+	56/104/2.615e − 01
BROWNAL (200)	Dai_Kou	22/42/1.004e − 02
	PRP+	-/-/-
BROWNALE (200)	Dai_Kou	1/1/9.500e − 05
	PRP+	1/1/1.070e − 04
BRYBND (5,000)	Dai_Kou	24/34/3.827e − 02
	PRP+	32/62/9.025e − 02
CHAINWOO (4,000)	Dai_Kou	223/361/2.337e − 01
	PRP+	271/480/4.458e − 01
CHNROSNB (50)	Dai_Kou	344/548/3.404e − 02
	PRP+	564/952/8.028e − 02
CRAGGLVY (5,000)	Dai_Kou	142/273/2.638e − 01
	PRP+	-/-/-
COSINE (1,000)	Dai_Kou	9/22/6.495e − 03
	PRP+	14/25/1.433e − 02
CURLY10 (10,000)	Dai_Kou	-/-/-
	PRP+	20,040/39,984/6.169e + 01
CURLY20 (10,000)	Dai_Kou	-/-/-
	PRP+	27,216/54,259/1.278e + 02
DIXMAANA (3,000)	Dai_Kou	10/12/5.625e − 03
	PRP+	16/27/2.274e − 02
DIXMAANB (3,000)	Dai_Kou	10/12/5.704e − 03
	PRP+	11/15/1.145e − 02
DIXMAANC (3,000)	Dai_Kou	12/15/6.271e − 03
	PRP+	14/21/1.697e − 02
DIXMAAND (3,000)	Dai_Kou	14/17/1.011e − 02
	PRP+	16/24/1.547e − 02
DIXMAANE (3,000)	Dai_Kou	85/123/4.520e − 02
	PRP+	80/152/8.792e − 02
DIXMAANF (3,000)	Dai_Kou	31/42/2.522e − 02
	PRP+	30/41/4.214e − 02
DIXMAANG (3,000)	Dai_Kou	29/40/2.873e − 02
	PRP+	27/35/2.557e − 02
DIXMAANH (3,000)	Dai_Kou	28/37/1.468e − 02
	PRP+	26/34/2.635e − 02
DIXMAANI (3,000)	Dai_Kou	124/186/6.319e − 02
	PRP+	124/239/1.124e − 01
DIXMAANJ (3,000)	Dai_Kou	36/52/2.502e − 02
	PRP+	31/43/3.019e − 02
DIXMAANK (3,000)	Dai_Kou	34/48/2.063e − 02
	PRP+	28/37/2.864e − 02
DIXMAANL (3,000)	Dai_Kou	29/40/1.661e − 02
	PRP+	30/40/3.369e − 02
DIXMAANM (3,000)	Dai_Kou	104/154/6.135e − 02
	PRP+	157/305/1.407e − 01
DIXMAANN (3,000)	Dai_Kou	63/93/3.813e − 02
	PRP+	98/164/8.303e − 02
DIXMAANO (3,000)	Dai_Kou	59/86/2.737e − 02
	PRP+	80/130/7.730e − 02
DIXMAANP (3,000)	Dai_Kou	56/77/3.176e − 02
	PRP+	72/111/6.704e − 02
DIXON3DQ (10,000)	Dai_Kou	620/945/5.557e − 01
	PRP+	1,467/2,933/2.524e + 00
DMN15103LS (99)	Dai_Kou	119/206/1.417e + 00
	PRP+	39/106/1.053e + 00
DMN15333LS (99)	Dai_Kou	80/171/1.143e + 00
	PRP+	-/-/-
DQDRTIC (5,000)	Dai_Kou	53/100/6.594e − 02
	PRP+	76/151/1.327e − 01
DQRTIC (5,000)	Dai_Kou	18/31/1.109e − 02
	PRP+	25/25/2.123e − 02
EDENSCH (1,000)	Dai_Kou	28/43/1.159e − 02
	PRP+	31/51/1.590e − 02
EG2 (1,000)	Dai_Kou	19/37/9.933e − 03
	PRP+	32/58/2.803e − 02
EIGENALS (2,550)	Dai_Kou	24,758/37,853/2.181e + 02
	PRP+	21,640/41,892/3.618e + 02
ENGVAL1 (1,000)	Dai_Kou	25/35/6.147e − 03
	PRP+	20/28/1.253e − 02
ERRINROS (50)	Dai_Kou	111/171 /1.860e − 02
	PRP+	25,995/48,312/3.756e + 00
ERRINRSM (50)	Dai_Kou	419/805/4.634e − 02
	PRP+	-/-/-
EXTROSNB (1,000)	Dai_Kou	652/1,063/1.300e − 01
	PRP+	906/1,611/2.639e − 01
FLETBV3M (5,000)	Dai_Kou	115/263/4.331e − 01
	PRP+	33/61/1.482e − 01
FLETCBV2 (5,000)	Dai_Kou	1/1/1.099e − 03
	PRP+	1/1/1.283e − 03
FMINSRF2 (5,625)	Dai_Kou	251/386/2.966e − 01
	PRP+	338/567/6.821e − 01
FREUROTH (5,000)	Dai_Kou	191/331 /2.437e − 01
	PRP+	75/133/1.523e − 01
GENHUMPS (5,000)	Dai_Kou	9,378/20,870/3.155e + 01
	PRP+	10,235/17,320/3.504e + 01
GENROSE (1,000)	Dai_Kou	3,054/4,706/7.083e − 01
	PRP+	4,947/8,388/1.792e + 00
HYDC20LS (99)	Dai_Kou	2,541/3,952/4.016e − 01
	PRP+	-/-/-
INDEF (5,000)	Dai_Kou	-/-/-
	PRP+	-/-/-
INDEFM (1,000)	Dai_Kou	-/-/-
	PRP+	628/1,271/5.722e − 01
JIMACK (3,549)	Dai_Kou	716/1,098/4.231e + 01
	PRP+	401/725/4.284e + 01
LIARWHD (5,000)	Dai_Kou	50/150/8.031e − 02
	PRP+	124/223/1.945e − 01
MANCINO (100)	Dai_Kou	8/17/5.880e − 02
	PRP+	31/59/2.788e − 01
MODBEALE (10,000)	Dai_Kou	371/738/1.879e + 00
	PRP+	-/-/-
MOREBV (5,000)	Dai_Kou	1/1/5.170e − 04
	PRP+	1/1/7.230e − 04
MSQRTALS (1,024)	Dai_Kou	749/1,148/1.534e + 00
	PRP+	520/969/1.854e + 00
MSQRTBLS (1,024)	Dai_Kou	783/1,196/1.639e + 00
	PRP+	681/1279/2.391e + 00
NCB20 (5,010)	Dai_Kou	365/688/1.466e + 00
	PRP+	148/248/8.941e − 01
NCB20B (5,000)	Dai_Kou	98/172/3.661e − 01
	PRP+	77/131/4.434e − 01
NONCVXU2 (5,000)	Dai_Kou	1,159/1,751/1.945e + 00
	PRP+	4,582/8,610/1.396e + 01
NONCVXUN (5,000)	Dai_Kou	1,247/1,887/2.110e + 00
	PRP+	9,929/18,942/3.063e + 01
NONDIA (5,000)	Dai_Kou	13/23/1.189e − 02
	PRP+	54/103/8.099e − 02
NONDQUAR (5,000)	Dai_Kou	66/129/5.082e − 02
	PRP+	139/202/1.238e − 01
OSCIGRAD (10,000)	Dai_Kou	31/44/5.616e − 02
	PRP+	-/-/-
OSCIPATH (500)	Dai_Kou	30/78/6.678e − 03
	PRP+	-/-/-
PENALTY1 (1,000)	Dai_Kou	18/28/4.520e − 03
	PRP+	-/-/-
PENALTY2 (200)	Dai_Kou	112/164 /2.145e − 02
	PRP+	173/304/5.560e − 02
PENALTY3 (200)	Dai_Kou	-/-/-
	PRP+	-/-/-
POWELLSG (5,000)	Dai_Kou	118/225/7.709e − 02
	PRP+	147/260/1.233e − 01
POWER (10,000)	Dai_Kou	22/25/1.965e − 02
	PRP+	-/-/-
QUARTC (5,000)	Dai_Kou	18/31/9.852e − 03
	PRP+	25/25/2.080e − 02
SCHMVETT (5,000)	Dai_Kou	38/68/1.145e − 01
	PRP+	33/63/1.478e − 01
SENSORS (100)	Dai_Kou	-/-/-
	PRP+	32/65/4.099e − 01
SINQUAD (5,000)	Dai_Kou	117/270/2.988e − 01
	PRP+	182/342/5.408e − 01
SPARSINE (5,000)	Dai_Kou	875/1348/1.708e + 00
	PRP+	-/-/-
SPARSQUR (10,000)	Dai_Kou	21/22/4.845e − 02
	PRP+	16/16/6.262e − 02
SPMSRTLS (4,999)	Dai_Kou	136/219/1.742e − 01
	PRP+	161/278/3.338e − 01
SROSENBR (5,000)	Dai_Kou	26/63/2.904e − 02
	PRP+	33/57/4.532e − 02
SSBRYBND (5,000)	Dai_Kou	6,337/9,751/9.184e + 00
	PRP+	-/-/-
SSCOSINE (5,000)	Dai_Kou	-/-/-
	PRP+	-/-/-
TESTQUAD (5,000)	Dai_Kou	5,068/7,734/1.948e + 00
	PRP+	1,624/3,247/9.661e − 01
TOINTGOR (50)	Dai_Kou	131/195/1.998e − 02
	PRP+	105/180/2.060e − 02
TOINTGSS (5,000)	Dai_Kou	18/37/2.997e − 02
	PRP+	14/27/2.830e − 02
TOINTPSP (50)	Dai_Kou	142/268/2.158e − 02
	PRP+	115/194/2.190e − 02
TOINTQOR (50)	Dai_Kou	43/64/7.463e − 03
	PRP+	41/81/9.627e − 03
TQUARTIC (5,000)	Dai_Kou	35/103/4.848e − 02
	PRP+	68/120/7.646e − 02
TRIDIA (5,000)	Dai_Kou	1,633/2,491/7.701e − 01
	PRP+	628/1,255/5.693e − 01
VARDIM (200)	Dai_Kou	18/18/1.765e − 03
	PRP+	-/-/-
VAREIGVL (50)	Dai_Kou	19/29/4.227e − 03
	PRP+	23/39/6.727e − 03
WOODS (4,000)	Dai_Kou	36/67/3.083e − 02
	PRP+	22/28/2.143e − 02

**Table 2:** **Numerical results for some boundary value problems**

**Name (Dim)**	**Method**	**Iter/Ng/CPU**
Function2 (10,000)	Dai_Kou	12/27/1.266e − 02
	PRP+	12/23/1.529e − 02
Function6 (10,000)	Dai_Kou	1/1/5.010e − 04
	PRP+	1/1/4.399e − 04
Function8 (10,000)	Dai_Kou	12/16/4.678e − 02
	PRP+	10/17/7.151e − 02
Function12 (10,000)	Dai_Kou	10/21/1.206e − 02
	PRP+	10/19/1.227e − 02
Function13 (10,000)	Dai_Kou	222/330/2.044e − 01
	PRP+	346/691/5.704e − 01
Function14 (10,000)	Dai_Kou	12/17/4.554e − 02
	PRP+	9/11/4.912e − 02
Function18 (10,000)	Dai_Kou	1/1/8.588e − 04
	PRP+	1/1/7.632e − 04
Function19 (10,000)	Dai_Kou	9/14/1.084e − 02
	PRP+	8/12/1.551e − 02
Function20 (10,000)	Dai_Kou	1/1/7.464e − 04
	PRP+	1/1/9.391e − 04
Function21 (10,000)	Dai_Kou	75/81/5.441e − 02
	PRP+	-/-/-
Function22 (10,000)	Dai_Kou	13/21/1.300e − 02
	PRP+	12/21/1.580e − 02
Function24 (10,000)	Dai_Kou	5/7/7.387e + 00
	PRP+	6/10/1.609e + 01
Function25 (10,000)	Dai_Kou	12/22/2.008e − 02
	PRP+	16/26/4.658e − 02
Function26 (10,000)	Dai_Kou	258/387/1.890e − 01
	PRP+	345/689/4.391e − 01
Function27 (10,000)	Dai_Kou	143/212/1.285e − 01
	PRP+	171/341/2.837e − 01
Function29 (10,000)	Dai_Kou	2,211/3,355/6.638e + 00
	PRP+	8,150/16,299/4.633e + 01
Function31 (10,000)	Dai_Kou	1/1/5.388e − 04
	PRP+	1/1/9.083e − 04

## Conclusions

In this paper, we discussed the improved Dai-Kou type conjugate gradient methods only using gradient information. They inherited the advantages of the Dai-Kou type conjugate gradient methods for solving the unconstrained minimization problems, but had broader application scope. Moreover, the problem considered in this paper can be viewed as the nonlinear equation

46 $$ F(x)=0 $$

with $F=g$. While the convergence analysis of this paper needed some assumptions of the function *f* whose gradient is *g*, our further investigation is to avoid the function *f* and to solve general nonlinear equation (46) using different strategies from those of this paper and literature [23–25].
